# Retraction notice

**DOI:** 10.1016/j.jaci.2021.02.002

**Published:** 2021-04

Retraction notice to Skin barrier dysfunction measured by transepidermal water loss at 2 days and 2 months predates and predicts atopic dermatitis at 1 year

J Allergy Clin Immunol 2015;135:930-935.e1

Maeve Kelleher MB ^a^, Audrey Dunn-Galvin PhD ^a^, Jonathan O'B, Hourihane DM ^a b^, Deirdre Murray MD ^a b^, Linda E. Campbell BSc ^c^, W.H. Irwin McLean DSc, FRS ^c^, Alan D. Irvine MD ^b d e^

^a^ Paediatrics and Child Health, University College Cork, Cork, Ireland

^b^ National Children's Research Centre, Dublin, Ireland

^c^ Dermatology and Genetic Medicine, University of Dundee, Dundee, United Kingdom

^d^ Paediatric Dermatology, Our Lady's Children's Hospital, Dublin, Ireland

^e^ Clinical Medicine, Trinity College Dublin, Dublin, Ireland

This article has been retracted: please see Elsevier Policy on Article Withdrawal (http://www.elsevier.com/locate/withdrawalpolicy).

This article has been retracted at the request of the Editors after the authors informed them of substantive errors in the reported methods, results and conclusions that affect the reliability of the results.

The authors have provided the following statement regarding the retraction of the two papers:

“In the process of establishing other studies, we re-examined our data relating to neonatal transepidermal water loss (TEWL). We were unable to replicate the published findings that TEWL quartiles at 2 days and 2 months predicted atopic dermatitis (AD) at 12m (1) or food allergy at 2 years (2). We sought independent statistical advice, giving that person unrestricted access to our data. Neither we nor the independent assessor - with whom we worked openly - have been able to replicate the findings. Furthermore we cannot identify the coding or calculation error for the derived variables that led to this problem. These problems are due to human error, and we affirm there is no evidence of fraudulent practice or intention on anyone’s part to mislead the Journal or the wider scientific community.
The AD-predictive link created in our modelling between *FLG* status and TEWL(1) is an error (tables 5 and 6). Figures 1 and 2 in the first paper (1), which are cited and presented widely, are based on erroneous analysis and need to be withdrawn. The independent, de novo analysis shows that the TEWL modelling data as published cannot stand for the whole cohort, though it does hold for a small subset (n=113) for which we have data about use of emollients in the early neonatal period. We believe a transcription error occurred in the data handling process meaning the findings for this subset were accidentally applied to the entire dataset.
There are several aspects of both papers that are independent of the TEWL modelling and we stand over these. We remain confident of the integrity of the descriptive data in both papers. These include the evolution of TEWL in the first year of life and the description of a positive relationship between changes in TEWL from birth to 6 months and TEWL findings at these time points (1). We have revalidated all the demographic and food allergy prevalence data and the association of *FLG* status and any AD/eczema in the first 2 y of life with food sensitization and food allergy at 2y, which support our own and others’ previous results (2).
We acknowledge that both papers are now widely cited, on the basis of the predictive values that we cannot replicate. Therefore, despite our belief in the integrity of the other data published, we must request full retraction of both papers.”

1. Kelleher M, Dunn-Galvin A, Hourihane JO’B, Murray D, Campbell LE, McLean WHI, Irvine AD. Skin Barrier Dysfunction measured by Transepidermal Water Loss (TEWL) at 2 days and 2 months Predates and Predicts Atopic Dermatitis at 1 year. J Allergy Clin Immunol 2015; 135 (4) 930-935. https://doi.org/10.1016/j.jaci.2014.12.013

2. Kelleher M, Dunn-Galvin A, Gray C, Murray D, Kiely M, Kenny L, McLean WHI, Irvine AD Hourihane JO’B. Skin barrier impairment at birth predicts food allergy at two years of age. J Allergy Clin Immunol April 2016 https://doi.org/10.1016/j.jaci.2015.12.1312

